# Effects of stepped speech rehabilitation and psychological intervention on speech disorders and cognitive function in Parkinson disease patients

**DOI:** 10.1097/MD.0000000000036420

**Published:** 2023-12-29

**Authors:** Liping Wang, Chengyan Qi, Minmin Gu, Min Yan, Qinde Qi

**Affiliations:** a Department of Neurology, Jinan City People’s Hospital, Jinan, 271199, Shandong, China.

**Keywords:** cognitive functioning, nursing outcomes, psychological interventions, rehabilitative exercise care, speech patients with Parkinson disease, stepwise

## Abstract

To examine the impact of stepwise speech rehabilitation exercise therapy in the treatment of patients with Parkinson speech problems under psychological intervention on clinical results and cognitive functioning. Parkinson speech disorder patients who met the inclusion criteria were selected and divided into a control group and an observation group for training respectively. The control group used conventional nursing methods, including training in orofacial movement, vocalization, pitch, volume and breath control. The observation group used stepwise speech rehabilitation exercise intervention combined with psychotherapy nursing programme. In the statistical analysis, independent sample t-test and chi-square test were used to test the significance of the data processing methods. In the statistical analysis of baseline functional level (*P* > .05). The difference was not statistically significant. After 7 weeks of training, the mFDA level and speech intelligibility increased in both the observation and control groups. From the situation analysis of “modified drinking test” and the comparison of UPDRS-I scores, it can be seen that dysphagia and Parkinson dysphasia were reduced in both groups after training. The observation group spontaneous speech dimension was greater than the control group by around 0.07 in the aphasia comparison. Both groups displayed an upward trend in their MMSE and Montreal Cognitive Assessment (MoCA) when measuring cognitive function; the evaluation of P300, constructive function, and quality of life revealed this. The observation group P300 potential score was 0.13 points higher than that of the control group. The therapeutic training of stepped speech rehabilitation exercise care combined with psychological intervention has significant nursing effects on patients with Parkinson disease speech disorders, and the patients’ cognitive functions have been effectively improved.

## 1. Introduction

Parkinson disease (PD) is a progressive, chronic neurological condition characterized by symptoms such as tremors, muscle rigidity, and dyskinesia.^[1]^ Alongside these primary symptoms, individuals with PD frequently experience various degrees of speech disorders (SD), which can manifest as slurred speech, slowed speech, and muffled voice.^[2]^ These speech disorders not only disrupt social communication and quality of life but also impact cognitive function (CF). Research has shown that PD-related mild cognitive impairment is a risk factor for the development of dementia, with a combined prevalence of mild cognitive impairment observed in 40% of a total sample of 7053 PD patients.^[3]^ Currently, the primary rehabilitative approaches for Parkinson speech disorder (PSD) include speech rehabilitation exercises and psychological interventions (PI).^[4]^ In a study exploring the impact of PD on verbal cognition, Groote team discovered that PD patients allocate more cognitive resources to support speech processing in noisy environments.^[5]^ As a result, speech rehabilitation exercises can be utilized to enhance articulation clarity and speech fluency through techniques like articulation, intonation, and speech rate practice.^[6]^ PI, on the other hand, assists patients in regulating their emotions, boosting self-confidence, and improving social skills through cognitive-behavioral therapy and other strategies.^[7]^ Nevertheless, there is a relative scarcity of research regarding the effects of stepped speech rehabilitation exercise nursing (SSREN) combined with PI on the care of PSD patients and their CF. Consequently, this study aims to introduce novel ideas and methods for the rehabilitation nursing of PSD patients through the use of SSREN combined with PI, thereby enriching existing nursing practices and providing a scientific basis for clinical implementation. This research employs a randomized controlled trial to examine the impact of SSREN combined with PI on the care of PSD patients and their CF. The study involves recruiting a specific number of PSD patients and randomly assigning them to experimental and control groups to investigate the outcomes.

## 2. Objects and methods

### 2.1. Research objects

This study has been approved by the Ethics Committee of Jinan City People Hospital. A total of 96 PSD patients admitted to a hospital between July 2021 and July 2022 were chosen as the study subjects and were randomly assigned to a control group and an experimental group, with 48 patients in each group, in order to better conduct this experiment. The admission criteria for the PSD patients selected for this study were as follows: Patients who were over 40 years of age and had been diagnosed with PD by a physician. Presence of significant SD, including articulation slurring, slowing of speech, and slurring of voices. Complete self-care or needing mild help with daily activities. Willingness to participate in the treatment of speech rehabilitation exercises and PI, and the ability to follow the treatment plan for the appropriate training and intervention. The following exclusion criteria applied to the study participants: Patients who also suffered from other serious neurological or psychiatric diseases, such as stroke, Alzheimer disease, etc. Patients with abnormal thyroid function or other endocrine system diseases that may affect the recovery of speech function. Patients with serious cognitive impairment or intellectual disability who are unable to participate effectively in speech rehabilitation exercises and PI. Patients with other serious physical diseases or systemic diseases, such as heart disease, lung disease, etc, that preclude speech rehabilitation exercise and PI treatment. Patients who have received similar speech rehabilitation exercise and PI treatment that may interfere with the study results. Patients with severe hearing impairment that precludes them from receiving speech rehabilitation exercise and PI. Patients with severe hearing impairment that precludes them from receiving speech rehabilitation exercise and PI. Before implementing the study, ethical principles were followed to secure informed consent from all patients, ensuring the privacy and confidentiality of their personal information. Table 1 provides essential demographic information about the study participants.

**Table 1:** Basic information of the study subjects.

Item		Intervention group (n = 48)	Control group (n = 48)	T/x^2^	*P*
Gender	Male	25	24	0.342	.895
	Female	23	24	0.457	.876
Age (yr)		67.28 ± 7.35	67.36 ± 7.19	0.213	.925
Marital status	Be married	30	29	0.521	.756
	Spinster	10	11	0.295	.798
	Divorce	5	4	0.386	.824
	Be bereaved of one spouse	3	4	0.299	.779
Educational level	Primary school	12	13	0.218	.858
	Junior high school	11	12	0.269	.799
	Senior high school	13	12	0.197	.861
	College or above	12	11	0.288	.748
Duration of disease (yr)		5.11 ± 1.25	5.23 ± 1.58	0.214	.571

Table 1 provides an overview of the demographic characteristics of the study participants in both the intervention and control groups. The data shows that there were no significant differences in the mean age, mean duration of the disease, marital status, or literacy levels between the 2 groups. In the intervention group, the mean age of participants was 67.28 ± 7.35 years, and the mean duration of the disease was 5.11 ± 1.25 years. In comparison, the control group had a mean age of 67.36 ± 7.19 years and a mean disease duration of 5.23 ± 1.58 years. These values were quite similar between the 2 groups. Regarding marital status, both groups had similar proportions of married, single, divorced, and widowed individuals. In the intervention group, there were 30 married participants, 10 single participants, 5 divorced participants, and 3 widowed participants. Similarly, the control group had 29 married participants, 11 single participants, 4 divorced participants, and 4 widowed participants. In terms of literacy, the distribution of educational levels was almost identical between the 2 groups. In the intervention group, there were 12 participants with primary and secondary education, 11 with junior high school education, 13 with high school education, and 12 with college or higher education. The control group had 13 participants with primary and secondary education, 12 with junior high school education, 12 with high school education, and 11 with college or higher education. Overall, these findings indicate that the demographic characteristics of the participants in both groups were well-matched, allowing for a more robust comparison of the intervention effects on speech disorders in Parkinson disease.

### 2.2. Research methods

Depending on the severity of the patients’ SD, the control group used conventional care, including training in orofacial movement, vocalization, pitch, volume and respiratory control.^[8]^ Each training time was 60 minutes, which was performed on alternate days, and the training time was 12 weeks. The observation group used stepwise speech rehabilitation exercise intervention combined with psychotherapy nursing programme. Firstly, caregivers influence patients’ negative emotions through non-verbal forms, such as eyes, expressions and behaviors, so that patients are in the best psychological state to participate in rehabilitation care. We should be in-depth and meticulous, carefully observe the changes of the condition and the psychological activities of the patient, master the formation of the psychological characteristics of the patient and the law of psychological activities, and carry out psychological nursing with a target. Take the initiative to communicate with patients, establish a pleasant working and living atmosphere for them, and make patients feel at ease. Patients should be actively encouraged to create a good treatment and recuperation environment for them. For patients with doubts, paper and pen are provided for patients to write down their problems and timely explanations are given. Secondly, professionally trained rehabilitation nursing staff conduct speech rehabilitation training for patients. The training included asking the patient to produce a continuous cause with maximum duration, instructing the patient to inhale deeply and prolonging the duration of the cause as much as possible. At the same time, the patient was instructed to amplify the pitch of the voice and to pronounce it at a low pitch, and the training was performed 10 to 12 times a day. Finally, according to the severity of the patient SD, stepwise voice intensity training was performed. Training was performed in the order from reading words and phrases aloud to reading sentences, reading and daily conversation, and the duration and frequency of training were the same as that of the control group.

### 2.3. Observation indicators

Before speech training, after 1 week of training, after 2 weeks, after 3 weeks, after 4 weeks, after 5 weeks, after 6 weeks, and after 7 weeks, a person was assigned to collect and analyze the information of the 2 groups of patients. The main observational indicators included the Modified Frenchay Dysarthria Assessment (mFDA) and the Speech Clarity Test, which were used to assess speech dysarthria.^[9]^ Meanwhile, Modified-Water-Swallow-Test was used to observe the effect of training of the organ of sound formation on swallowing function in patients with PD.^[10]^ In addition, the Unified Parkinson Disease Rating Scale (UPDRS)-I was used to assess the effects of training on the patients’ daily living status, mood and intelligence-related conditions. With these ratings, the improvement effect of training on patients can be observed. In addition, the study used aphasia profiles (auditory comprehension, repetition, and spontaneous speech), Montreal Scale, Mini-Mental State Examination (MMSE), P300 latency and amplitude, and constructive functioning and quality of life to compare and analyze the effectiveness of nursing care and CF in patients with PD.

### 2.4. Statistical methods

Using statistical software SPSS version 23.0, all the data collected for this study were statistically evaluated. A paired samples t-test was used to determine the significance of the comparison of data before and after treatment, and an independent samples t-test was used to determine the significance of the comparison of measurement data between the 2 groups in cases where the measurement data were expressed using mean and standard deviation. In addition, in the comparison of count data, the chi-square test was used for significance testing. When the *P* < .05, there is a significant difference between the 2 groups of data.

## 3. Results

### 3.1. Comparison of baseline functional levels

In order to obtain the baseline functional level of the observation and control groups before training, the mFDA score, speech intelligibility, “Modified Drinking Test,” UPDRS-I level, phonological function, and quality of life scores of the 2 groups were compared and evaluated, and the results of the comparison of the baseline functional level of the 2 groups of patients before the experiment are shown in Table 2.

**Table 2:** Comparison of baseline functional level between the 2 groups before the experiment.

Group	Observation group	Control group	T/ x^2^	*P*
mFDA(Item)	13.10 ± 4.46	12.04 ± 5.07	−0.562	.577
Language articulation (%)	66.2 ± 17.60	71.2 ± 14.55.	−0.688	.495
Drinking water experiment pass number (Example)	5	3	0.115	.737
UPDRS-I(%)	3.30 ± 1.38	1.05 ± 1.67	0.543	.590
Articulation function A-level items (Items)	5.12 ± 4.25	5.14 ± 4.27	0.458	.487
PDQ39 Score (Points)	37.68 ± 10.13	37.72 ± 10.32	0.381	.613

As can be seen from Table 2, the P-values of mFDA, speech intelligibility, the number of passed drinking experiments, UPDRS-I, the number of items of the a-level of the conformational function and the PDQ39 scores of the patients of the 2 groups are all >0.05, which indicates that the differences in the indexes are statistically insignificant and comparable. Among them, the observation group scored 13.10 in mFDA and 66.4% in speech intelligibility, the mean mFDA score of the control group was 1.06 points lower than that of the observation group, and the mean score of speech intelligibility was 5 points higher than that of the observation group.

### 3.2. Comparison of mFDA levels and comparison of speech clarity

**Figure 1.**
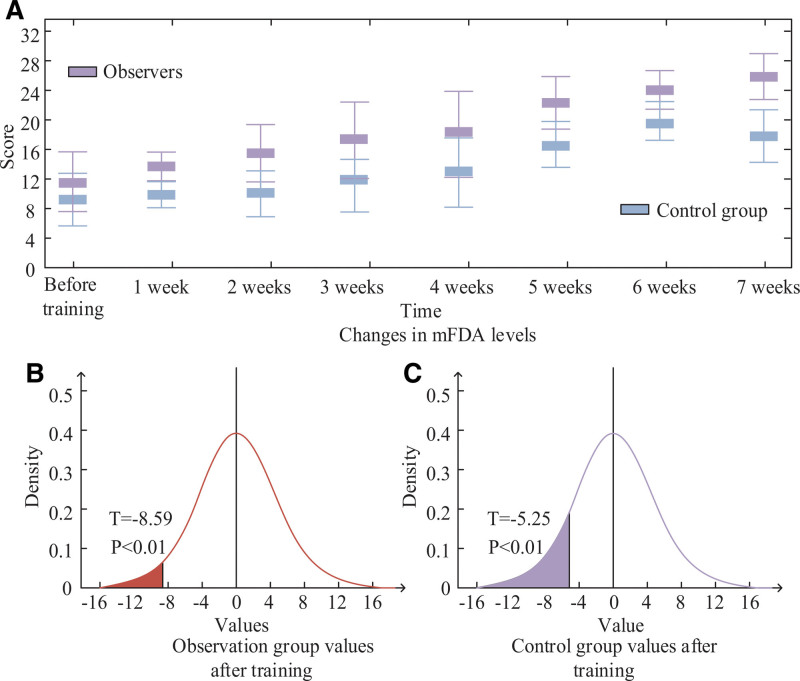
Changes in mFDA levels before and after training.

Figure 1 displays the changes in mFDA levels for both the observation group and the control group over the course of the comparison experiment. Observation Group (Intervention Group): The mFDA level in the observation group showed a significant improvement 7 weeks after training. It increased by approximately 12 points compared to the pre-training mFDA level. The *P* value for the post-training mFDA score was <.05, indicating statistical significance. This suggests that the speech rehabilitation exercises and psychological interventions had a positive impact on improving speech articulation and clarity in the observation group. Control Group: Similarly, the mFDA level in the control group also improved at the 7-week mark after training, showing an increase of about 8 points compared to the pre-training mFDA level. The *P* value for the post-training mFDA score was <.05, indicating statistical significance. While the control group experienced improvement, it was slightly less pronounced than the improvement observed in the observation group. Overall, both groups demonstrated significant improvements in mFDA levels after 7 weeks of training, suggesting that speech rehabilitation exercises and psychological interventions had a positive effect on speech articulation in Parkinson disease patients. However, the observation group, which received the combined intervention, showed a larger improvement compared to the control group. This indicates that the combination of SSREN (stepped speech rehabilitation exercise nursing) and PI (psychological interventions) may lead to greater improvements in speech clarity and fluency compared to speech exercises alone.

The level of speech intelligibility is one of the important factors to judge the effect of SSREN on the care of PSD patients. Therefore, the speech intelligibility of the control group and the observation group before and after training was compared and analyzed, and speech intelligibility was divided into 4 dimensions: hearing impairment degree, language ability, language familiarity, and lip-reading in training. These 4 dimensions affect speech articulation mainly from acoustic aspects, language aspects, and lip-reading aspects, and these 4 dimensions are the main factors affecting the speech articulation of Parkinson patients. As can be seen from Table 3, the overall difference in speech intelligibility between the 2 groups of research subjects before training is not significant. After 7 weeks of training, all 4 dimensions of the control group were improved, and the p-values before and after training were in the range of 0.03 to 0.04, which was significant; while the changes in the 4 dimensions of the observation group were more obvious, and their p-values before and after training were around 0.01, indicating that the changes were more significant in comparison with those of the control group.

**Table 3:** Comparison of language articulation between the 2 groups before and after training.

Group		Hearing loss (%)	Language ability (%)	Language familiarity (%)	Lip reading (%)
Control group	Before training	9.2 ± 1.60	66.2 ± 17.6	53.2 ± 15.7	36.2 ± 14.7
	After training	7.7 ± 3.20	84.2 ± 5.6	76.8 ± 9.9	42.1 ± 17.6
	T	-7.703	6.501	6.201	5.208
	*P*	0.04	0.03	0.04	0.03
Observation group	Before training	9.5 ± 7.50	67.8 ± 19.90	57 ± 18.60	32.4 ± 16.90
	After training	6.2 ± 1.90	87.2 ± 8.30	79.8 ± 13.80	49.8 ± 21.3
	T	−4.032	5.36	5.214	4.332
	*P*	.008	.013	.015	.017

### 3.3. Situation analysis of the “Improved Drinking Water Trial” and comparison of UPDRS-I scores

Table 4 presents the outcomes of assessing changes in swallowing abilities across 2 groups of patients following their respective training interventions. The study encompassed 20 participants for each group, and these individuals were categorized into 5 distinct levels representing different degrees of swallowing capacity. These levels were graded as follows: Grade 1: Denoting an absence of swallowing action, no choking, accompanied by respiratory changes, moist rale, and other associated reactions. Grade 2: Reflecting patients with swallowing movements, absence of choking, but notable respiratory changes. Grade 3: Indicating patients with swallowing movements, associated coughing, moist rale, yet no observed respiratory changes. Grade 4: Signifying patients displaying swallowing movements without choking, and the absence of both moist rale and respiratory changes. Grade 5: Representing patients exhibiting swallowing movements without choking, with no signs of moist rale or respiratory changes. The findings from Table 4 reveal the following trends: Observation Group (Intervention Group): Before their training intervention, the observation group comprised 3 patients with Grade IV swallowing difficulties and 1 patient with sub-heavy swallowing. Additionally, 1 patient exhibited Grade V dysphagia. Following the training program, the observation group displayed substantial improvements in their swallowing abilities, with no patients experiencing severe swallowing difficulties (Grade IV). Instead, 1 patient exhibited sub-heavy swallowing. Two patients still had Grade V dysphagia, and one had Grade IV dysphagia. Control Group: Prior to their training intervention, the control group included 2 patients with Grade V dysphagia and 1 patient with Grade IV dysphagia. After the training program, 1 patient continued to experience Grade V dysphagia, 1 had Grade IV dysphagia, and 2 patients exhibited Grade I dysphagia. To gauge the statistical significance of these changes, chi-square (x^2^) values were calculated for both groups after training. The x^2^ value for the observation group was 10.100, while that for the control group was 7.237. In the control group, the *P* values were <.05, indicating a statistically significant enhancement in swallowing ability following training. Conversely, in the observation group, the *P* value exceeded .05, signifying that the improvement in swallowing ability after training did not reach statistical significance. In summary, the control group experienced a statistically significant enhancement in swallowing ability after training, whereas the observation group did not exhibit a statistically significant improvement. These findings suggest that the specific intervention, involving stepped speech rehabilitation exercise nursing combined with psychological interventions, in the observation group may have had differing effects on swallowing ability compared to the control group, which received speech rehabilitation exercises alone.

**Table 4:** Comparison of the changes of “improved drinking water experiment” before and 7 wk after training between the 2 groups of patients (example).

Group		I	II	III	IV	V	x^2^	*P*
Observation group	Before training	8	6	3	3	1	10.100	.100
	After training	9	8	2	1	0		
Control group	Before training	8	5	4	1	2	7.237	.007
	After training	10	6	2	1	1		

**Figure 2.**
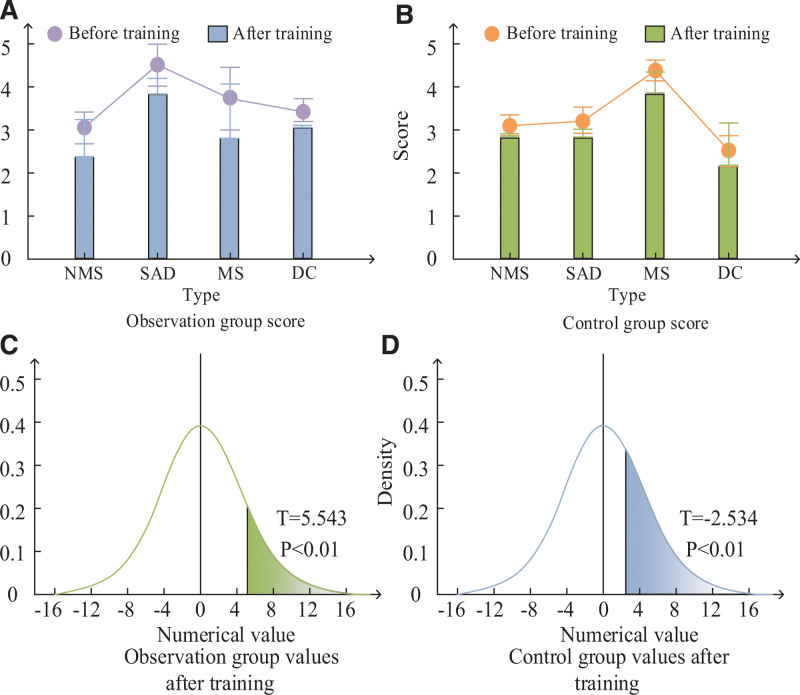
UPDRS-I score comparison. UPDRS-I = the Unified Parkinson Disease Composite Rating Scale.

The results of UPDRS-IL scale in both groups are shown in Figure 2. As can be seen in Figure 2, both the control and observation groups showed a decreasing trend in UPDRS-I scores after training. In comparison to the control group, the observation group demonstrated greater improvement in non-motor, activities of daily living (ADL), and motor symptoms. The observation group demonstrated decreases in these symptoms of about 0.5 more than the control group in motor symptoms, about 0.2 more than the control group in ADL symptoms, and more than 0.3 more than the control group in non-motor symptoms. It was also found that the *P* value of both groups after training was <.05 and hence statistically significant.

### 3.4. Comparison of aphasia

Using the aphasia set of examination criteria from the multidimensional aspects of naming, auditory comprehension, repetition, and spontaneous speech, the effect of the control group and the observation group are evaluated after Stepped Speech Rehabilitation Exercise Nursing (SSREN). Higher scores correspond to lower levels of aphasia, and the results of the comparison of aphasia evaluation are shown in Table 5. Table 5 demonstrates that, prior to training, the observation group scores in naming, listening comprehension, and repetition were lower than those of the control group, while their scores in the spontaneous speech dimension were roughly 0.07 higher than those of the control group. After 7 weeks of training, the scores of the observation group in all dimensions were improved and showed an upward trend. From the comparison of T and *P* values, the *P* values before and after training were <.05, indicating that the data were statistically significant.

**Table 5:** Comparison of aphasia before and after training between the 2 groups.

Group		Example number	Naming	Listening comprehension	Retelling	Spontaneous language
Control group	Before training	35	7.53 ± 1.32	6.34 ± 1.34	5.61 ± 2.31	18.35 ± 1.41
	After training	35	16.46 ± 1.24	17.42 ± 1.31	16.35 ± 1.22	35.46 ± 1.33
T		/	15.771	18.382	17.895	28.731
*P*		/	0.000	0.000	0.000	0.000
Observation group	Before training	35	6.48 ± 1.42	6.33 ± 1.23	5.58 ± 1.41	18.42 ± 1.39
	After training	35	15.23 ± 1.33	16.55 ± 1.31	15.53 ± 1.34	34.37 ± 1.41
T		/	20.614	23.119	22.452	35.576
*P*		/	0.000	0.000	0.000	0.000
T1		/	0.432	0.208	0.116	0.152
*P1*		/	0.667	0.834	0.905	0.877
T2		/	5.132	3.86	3.47	4.054
*P2*		/	0.000	0.000	0.001	0.000

### 3.5. Comparison of Montreal Scale and MMSE scale scores

The results of the Montreal Cognitive Assessment (MoCA) of the 2 groups are shown in Table 6. As shown in Table 6, the observation group improved 0.2 points in the MoCA dimension after 7 weeks of training, attention improved 0.05 points, delayed recall decreased 0.03 points, and the rest of the dimensions had a smaller increase. Overall, the observation group showed an upward trend in the MoCA after 7 weeks of training. The control group showed a small decrease in MoCA, visuospatial and executive ability, attention, verbal ability, delayed recall, and orientation after 7 weeks of training, with a 0.1 point increase in naming ability. The p-values of the scores of all dimensions except attention and abstraction ability were <0.05 for comparison after training, indicating that these dimensions were statistically significant between groups.

**Table 6:** Comparison of Montreal Scale scores before and after training between the 2 groups.

Item	Observation group		Control group		*P*
	Before training	After training	Before training	After training	
MoCA total score	20.1 ± 3.78	20.3 ± 3.78	20.12 ± 3.71	l9.16 ± 3.20	.000
Visual space and executive ability	2.73 ± 1.17	2.73 ± 1.19	2.67 ± 1.42	2.35 ± 1.18	.023
Naming capability	2.41 ± 0.72	2.41 ± 0.72	2.38 ± 0.81	2.48 ± 0.66	.027
Attention	3.92 ± 0.95	3.97 ± 0.93	4.05 ± 0.97	3.81 ± 1.02	.831
Language ability	2.16 ± 0.64	2.16 ± 0.64	2.12 ± 0.76	1.87 ± 10.81	.001
Abstract ability	1.12 ± 0.68	1.12 ± 0.66	1.13 ± 0.65	1.21 ± 0.68	.843
Delayed recall	2.56 ± 1.28	2.53 ± 1.28	2.67 ± 1.25	2.05 ± 1.06	.022
Directing force	5.16 ± 0.99	5.16 ± 0.99	5.24 ± 0.96	4.47 ± 0.93	.001

**Figure 3.**
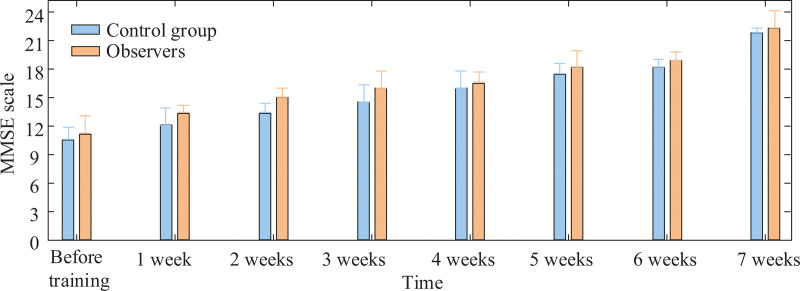
Comparison of MMSE Scale Scores.

The final assessment of the MMSE scale in both groups of patients is shown in Figure 3. Figure 3 shows the comparison of the MMSE scale scores of the 2 groups of patients before training and after 7 weeks of training. As can be seen in Figure 3, the scores of both groups of patients before training were around 10, indicating that both groups of patients had some degree of impairment in CF. During the training period, the scores of both groups of patients showed a gradual increase, indicating that SSREN had a certain effect on the treatment of cognition in both groups of patients, in which the observation group had a greater increase in score compared to the control group, with a score of more than 15 after 4 weeks of training, and the control group score was significantly improved after 6 weeks of training.

### 3.6. Comparison of P300, phonological function and quality of life between the two groups of patients

In order to understand the changes in the brain function of the 2 groups of patients before and after the treatment, the brain triggered potential, i.e., P300 was used to detect the response of the patient body regarding the sensory, visual and auditory pathways to the sensitivity or stimuli. P300 is a positive wave associated with cognitive function that can be detected on the scalp with a latency of about 300 milliseconds, and shorter latency and larger amplitude are associated with better cognitive performance. The control group and observation group were selected for experimental comparison and analysis and the final results are shown in Table 7. As can be seen from Table 7, the P300 latency of the observation group was at a moderate level compared to the score of the control group before training, and the P300 latency scores of the observation group were significantly shorter and smaller than those of the control group by 2 to 7 points after 4 and 7 weeks of treatment. In the comparison of P300 amplitude the observation group had the lowest score compared to the control group before training and 0.13 points higher than the control group after 7 weeks of training.

**Table 7:** Comparison of P300 latency and amplitude.

Time	Group	P300latent period (ms)	P300 amplitude(μv)
Before treatment	Con	325.65 ± 26.35	2.22 ± 0.98
	Observers	325.75 ± 26.13	2.22 ± 0.87
At 4 wk after treatment	Con	322.51 ± 23.76	2.26 ± 1.07
	Observers	318.23 ± 21.50	2.33 ± 1.12
At 7 wk after treatment	Con	319.46 ± 22.48	2.30 ± 1.34
	Observers	311.67 ± 24.12	2.43 ± 1.23

**Figure 4.**
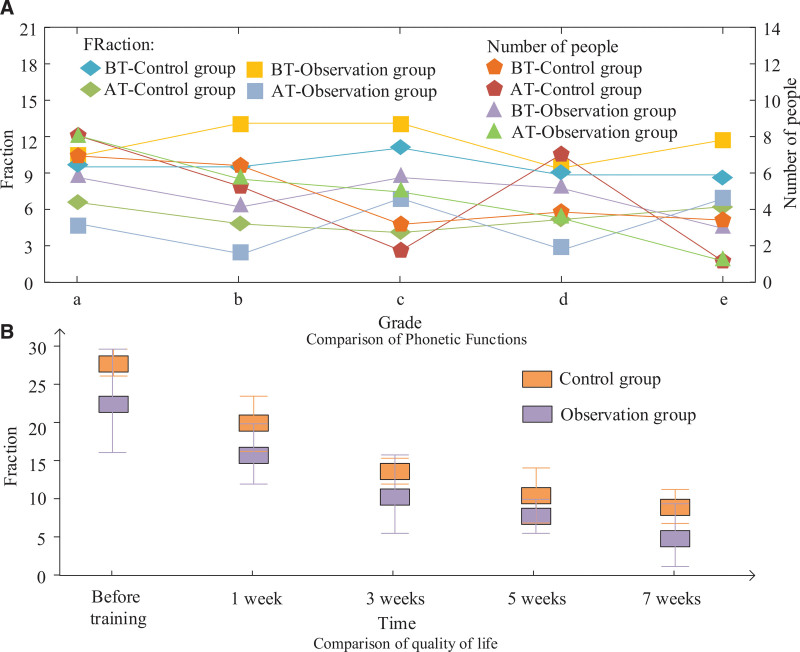
Changes in articulation function and quality of life of patients in the 2 groups.

In addition, in order to understand the changes in the patients’ conformational function and quality of life after treatment, the scale was evaluated in both groups. The severity of dysarthria was categorized into 5 levels, with normal as item a and e as severely impaired item. The grading standards are as follows: the lip movement is within normal range; the lip movement is somewhat weakened or excessive, occasionally leaking sound; poor lip movement, weak sound or undue plosive sound, and many parts of the lip shape do not meet the requirements; the patient has some lip movement, but no pronunciation can be heard; no movement of the lips was observed, even when trying to speak. The PD questionnaire (PDQ39) was chosen to evaluate the patients’ quality of life, with lower scores indicating a worse quality of life. The final results are shown in Figure 4. From Figure 4A, it can be seen that the severity of dysarthria was significantly reduced in both groups after training, and the number of patients with dysarthria severity was reduced, with a total of 4 fewer patients in the 2 groups of degree e, and an increase of 5 more patients in the 2 groups of degree a. From Figure 4B, it can be obtained that the quality of life score situation of both groups of patients showed a decreasing trend, indicating that the quality of life of both groups of patients improved after treatment, and the quality of life score of the observation group was about 5 points less than that of the control group after 7 weeks of training.

## 4. Discussion

Parkinson disease (PD) is a neurodegenerative disorder primarily characterized by motor dysfunction. However, PD patients often experience speech disorders (SD), including symptoms like dysarthria, slow speech, and weak voice, significantly impacting their daily communication.^[11]^ Stepped Speech Rehabilitation Exercise Nursing (SSREN) and Psychological Interventions (PI) are 2 commonly employed rehabilitation methods for Parkinson Speech Disorder (PSD). SSREN involves a series of physical therapy exercises aimed at enhancing articulation and speech fluency, encompassing oral muscle exercises, breathing techniques, and more. These exercises improve oral muscle strength and flexibility, leading to more precise and clear pronunciation. Breathing training helps patients control their breathing rhythm, promoting fluent speech and improved vocal volume and quality. PI, on the other hand, assists patients in managing negative emotions like anxiety and depression while enhancing Cognitive Function (CF) through psychotherapy and cognitive training.^[12]^ Given that psychological issues are prevalent in PD patients and can exacerbate SD and impact CF, psychotherapy plays a crucial role in improving SD.^[13]^ Despite the widespread use of SSREN and PI in PSD treatment, there is limited research on the combined application of these approaches and their impact on CF. To create a more effective PD patient rehabilitation plan, comprehensive studies are needed to assess the feasibility and effectiveness of this integrated treatment, ultimately improving patients’ quality of life and providing a scientific basis for clinical practice.

Research by Hall and others highlighted the significant impact of mFDA scores and speech intelligibility in PSD patients.^[14]^ In this study, the observation group showed a more substantial improvement in mFDA levels compared to the control group, with both groups experiencing noticeable mFDA improvement 4 weeks into the training. This suggests that the combined use of SSREN and PI has a more favorable impact on PSD patient rehabilitation, aligning with the findings of Hu team.^[15]^ Additionally, this study identified the suitability of the modified drinking test for severe dysphagia patients, echoing the results obtained by Chen et al in a similar drinking test study.^[16]^ To devise rational treatment plans for PD patients with speech impairments and evaluate the therapeutic progress of SSREN, the Unified Parkinson Disease Composite Rating Scale (UPDRS-I) was employed to assess and score both the observation and control groups. After training, both groups exhibited a decline in UPDRS-I scores, indicating a reduction in all PD symptoms, consistent with the findings of Wang team.^[17]^ Moreover, Zhang and colleagues, in their aphasia examination criteria study, observed increased scores in aphasic patients before and after training, aligning with the results of this study.^[18]^ In evaluating the cognitive therapeutic effects of SSREN on PSD patients, the MoCA scale was used to compare the observation and control groups. MoCA scores in the observation group exhibited improvement after 7 weeks of training, while the control group showed a slight decline in MoCA scores across various cognitive dimensions. Similar trends were identified in a study conducted by Hu team.^[15]^ To understand changes in brain function in both patient groups pre- and post-treatment, Brain Evoked Potentials, specifically P300, were employed to detect sensory, visual, and auditory pathway responses to sensitization or stimulation. Upon comparing P300 wave amplitudes, the observation group demonstrated more substantial post-training score improvements than the control group. This corresponds with findings in a study by Hosseini et al, which examined brain function changes in aphasic patients before and after treatment.^[19]^

In summary, this study underscores the clinical value of combining Stepped Speech Rehabilitation Exercise Nursing (SSREN) with Psychological Interventions (PI) for Parkinson Speech Disorder (PSD) patients. The training approach used in the observation group demonstrated superior efficacy compared to speech training alone in the control group, particularly in improving mFDA scores and speech intelligibility. However, it did not show a significant advantage in enhancing swallowing function. Consequently, the stepped speech rehabilitation exercise mode emerges as an efficient, safe, and well-accepted approach by patients. This research contributes valuable insights into effective and secure training methods and treatment tools for PSD patients. By exploring diverse training techniques and selecting optimal treatment approaches based on nursing effects and cognitive function outcomes, this study provides valuable resources for the treatment of PSD patients. One limitation of this study is the absence of equipment for detecting acoustic-related parameters during voice function training, leading to a lack of precise quantitative statistics on the efficacy of PSD patients’ voice intensity and fundamental frequency. This aspect could be further refined in subsequent studies.

## Author contributions

**Conceptualization:** Liping Wang, Chengyan Qi, Min Yan, Qinde Qi.

**Data curation:** Liping Wang, Chengyan Qi, Min Yan, Qinde Qi.

**Formal analysis:** Liping Wang, Chengyan Qi, Minmin Gu, Min Yan, Qinde Qi.

**Investigation:** Liping Wang, Chengyan Qi, Minmin Gu, Min Yan, Qinde Qi.

**Methodology:** Chengyan Qi, Minmin Gu, Min Yan, Qinde Qi.

**Supervision:** Minmin Gu, Min Yan, Qinde Qi.

**Writing – original draft:** Liping Wang.

**Writing – review & editing:** Liping Wang, Qinde Qi.
